# Histopathological analysis of respiratory muscles in patients with acute COVID-19 infection

**DOI:** 10.1007/s00441-025-03973-3

**Published:** 2025-05-05

**Authors:** Laura Steingruber, Simona Handtke, Franziska Schweiger, Stefan Schiele, Bruno Märkl, Marco Koch

**Affiliations:** 1 Anatomy and Cell Biology, Institute of Theoretical Medicine, Faculty of Medicine, University of Augsburg, Augsburg, Germany; 2 Centre for Interdisciplinary Health Research (CIHR), University of Augsburg, Augsburg, Germany; 3 Computational Statistics and Data Analysis, Institute of Mathematics, Faculty of Mathematics, Natural Sciences and Materials Engineering, University of Augsburg, Augsburg, Germany; 4 Institute of Pathology and Molecular Diagnostics, Faculty of Medicine, University of Augsburg, Augsburg, Germany

**Keywords:** COVID-19, Histopathology, Respiratory muscle degeneration (RMD), Ventilation support, Satellite cells

## Abstract

Coronavirus-disease 2019 (COVID-19) affects the respiratory system with high morbidity in elderly and comorbid patients. Acute COVID-19 infection (CI) primarily leads to respiratory failure, long-term effects on respiratory skeletal muscle however remain vague. Thus, histopathological marker expression of oxidative stress, inflammation, satellite cell activity, myosin fiber composition, and cellular senescence were analyzed in intercostal muscle and diaphragm to compare respiratory muscle degeneration (RMD) in deceased CI-positive and control patients. Beside CI, the impact of BMI, age, sex, ventilation status, and duration of hospitalization on RMD were evaluated. CI-positive patients exhibited higher numbers of regenerative stem cells, but no association between CI status and RMD was observed. Interestingly, ventilation support and lung-associated comorbidities had no effect on expression of RMD markers (*p* > 0.05). However, intercostal muscle showed BMI-dependent changes in expression of RMD markers, regardless of the CI status, with increased cytokine expression (*p* = 0.04), reduced antioxidative capacity (*p* = 0.05), and low stem cell prevalence (*p* = 0.02) in patients with high BMI. Moreover, elderly patients demonstrated increased oxidative stress (*p* = 0.001) and cell senescence (*p* = 0.03) independent of CI status. Notably, immobility drives muscle fiber transformation to Myosin ST (*p* = 0.03), since prolonged hospitalization correlated with muscle fiber type shift. Limitations included incomplete retrospective data collection and absence of adequate samples for molecular analyses. Together, RMD is influenced by BMI, age and immobility rather than the CI status alone. Future studies including larger cohorts, molecular analyses, and evaluation of patient data in addition to CI status alone, will further support meaningful analyses and interpretation of RMD and its impact on post CI recovery.

**Supplementary Information:**

The online version contains supplementary material available at 10.1007/s00441-025-03973-3.

## Introduction

Epidemiological analyses of Coronavirus-disease 2019 (COVID-19) reveal a high morbidity particularly in elderly patients and a severe course of the disease in predisposed subjects suffering from comorbidities such as obesity (Gao et al. 2021; Suwono et al. 2023; Zhang et al. 2023). Respiratory COVID-19 infection (CI) primarily leads to inflammatory and pneumonia-like symptoms (Rivera et al. 2023; Xu et al. 2020). Patients with acute CI majorly suffer from hypoxemia with dyspnea and require high-flow nasal oxygen or in severe cases mechanical ventilation (Butler et al. 2023; Obradović et al. 2023).

Beyond acute CI-associated symptoms, long-term persistence of dyspnea not only is caused by a functional impairment of the pulmonary system, but also CI-dependent pathophysiological processes in respiratory muscles, such as intercostal muscles and diaphragm, are discussed as underlying mechanisms (Ali & Kunugi 2021; Farr et al. 2021). In this, disturbance of the neuromuscular transmission, local cytokine release and other inflammatory events, increase in muscle catabolism and myopathy are associated with respiratory muscle degeneration (RMD) in CI patients (Pescaru et al. 2022). However, little is known about pathophysiological long-term changes in skeletal muscle tissue with special regard to intramural stem cells, known as satellite cells, which accomplish myogenesis to form novel myofibers (Schmidt et al. 2019).

A current therapeutic approach to treat long-term consequences of CI such as persistent breathing difficulties is to improve or restore the full function of the respiratory muscles (Bissett et al. 2020). In this context, our overall goal was to use histopathological investigations to determine how satellite cell-associated regulatory processes in respiratory muscles are affected by CI. These findings might be considered as a novel starting point for future therapy options to improve regeneration of the respiratory muscles. To include potential additional effectors beside CI, such as BMI, age, sex, ventilation status, and duration of the hospitalization, we have included biographical data and clinical findings in our comprehensive histopathological analysis on CI-associated RMD.

## Material and methods

### Acquisition of sample metadata

In-clinic patients deceased between March and September 2022 were included and pathologically examined at the Institute of Pathology and Molecular Diagnostics of University Hospital Augsburg. The composition of the collective was subject to an ethics vote (project number 22–0244, Ethics Committee of Ludwig-Maximilians-Universität München, Munich, Germany). Equal distribution of patients suffering from acute CI against CI-negative controls was considered. Corresponding population analysis (Table 1) revealed a mean age of 72 years (range 56–86) comprising 18 male and 13 female individuals. The group was divided into 16 CI-positive (Table 1, left column) and 15 CI-negative (Table 1, right column) patients. Group of CI-positive patients, of which 75% were male, displayed a median age of 66 years and a mean BMI of 29 kg/m2 with a standard deviation (SD) of 6.8 kg/m^2^ (men: 28 kg/m^2^, SD = 3.3 kg/m^2^; women: 32 kg/m^2^, and SD = 13 kg/m^2^). The CI-negative patient group contained 6 men (40%), showing a median age of 77 years and a mean BMI of 30.6 kg/m^2^ with a SD of 5 kg/m^2^ (men: 31 kg/m^2^, SD = 3.7 kg/m^2^; women: 30 kg/m^2^, and SD = 6 kg/m^2^). The BMI graded according to sex and CI status was associated with an obese to adipose degree 1 status (Schienkiewitz et al. 2022) in all calculations. A lung-associated comorbidity was documented in 7 CI-positive and 2 CI-negative patients. Determined duration of hospitalization of CI-positive patients displayed a median value of 7 days, in CI-negative patients 3 days.

**Table 1:** Statistical analysis of metadata derived from patients graded according to the status of CI. The following variables were evaluated: BMI, age, sex, times of COVID-19 vaccination, duration of hospitalization, and lung-associated comorbidity

**Characteristics**	**CI-positive**^**1**^**,** ***N*** **= 16**	**CI-negative**^**1**^**,** ***N*** **= 15**
BMI	MW: 29.2 (± 6.8), median: 29.0	MW: 30.6 (± 5.1), median: 30.0
Unknown	1	2
Age	MW: 71 (± 10), median: 66	MW: 74 (± 10), median: 77
Sex		
Male	12 (75%).	6 (40%).
Female	4 (25%).	9 (60%).
Times of COVIF-19 vaccination		
2x	2 (14%).	0 (0%).
3x	4 (29%).	6 (86%).
4x	1 (7.1%).	0 (0%).
Unvaccinated	7 (50%).	1 (14%).
Unknown	2	8.
Duration of hospitalization	MW: 9 (± 8), median: 7	MW: 9 (± 12), median: 3
Unknown	0	3
Lung-associated comorbidity		
Yes	7 (44%).	2 (13%).
No	9 (56%).	13 (87%).

### Acquisition of sample material

Tissue samples of intercostal muscle and diaphragm were prepared at the Institute of Pathology and Molecular Diagnostics of the University Hospital Augsburg (project number 22–0244, Ethics Committee of Ludwig-Maximilians-Universität München, Munich, Germany). Muscle tissue was further processed as formalin-fixed, paraffin-embedded samples for diagnostic analysis. For immunohistochemical analysis, 2-mm-thick sections were prepared.

###  Applied antibodies

All antibodies used in the present study are listed in Table 2.

**Table 2:** List of antibodies for immunohistochemical staining with information about producer, product number, host, clonality, conjugate, and applied dilution

**Primary antibodies**			
Target	Company, product number	Host, clonality	Dilution
ALDH1 A1	Abcam, Cambridge, UK; ab131068	Rabbit polyclonal	1:800
ALDH1 A3	Abcam, Cambridge, UK; ab129815	Rabbit polyclonal	1:200
CD56/NCAM1	Thermo Scientific, Munich, Germany; MA1-35,249	Mouse monoclonal	1:200
GLB1	Thermo Scientific, Munich, Germany; PA5-64,417	Rabbit polyclonal	1:800
IL-1 alpha	Thermo Scientific, Munich, Germany; PA5-85,366	Rabbit polyclonal	1:500
IL-6	Thermo Scientific, Munich, Germany; PA1-26,811	Rabbit polyclonal	1:400
Pax7	Thermo Scientific, Munich, Germany; PA1-117	Rabbit polyclonal	1:200
SelK	Abcam, Cambridge, UK; ab121276	Rabbit polyclonal	1:100
Myosin slow (ST)	Sigma-Aldrich, St. Louis; USA; M8421	Mouse monoclonal	1:800
Myosin fast (FT)	Sigma-Aldrich, St. Louis; USA; M4276	Mouse monoclonal	1:800
**Secondary antibodies**			
Target	Company, product number	Conjugate	Dilution
Goat anti-Mouse IgG (H + L)	Vector Laboratories, Newark, USA; BA-9200–1.5	biotinylated	1:400
Goat anti-Rabbit IgG (H + L)	Vector Laboratories, Newark, USA; BA-1000–1.5	biotinylated	1:400

### Immunohistochemistry

Sections were deparaffinized followed by epitope unmasking in pH 6.0 citrate buffer at 95° for 30 min. After incubating with endogenous peroxidase, the slides were quenched with 1.5% H_2_O_2_ and blocked in a mixture of blocking buffer (1 × phosphate-buffered saline (PBS); Thermo Fisher Scientific), 1% bovine serum albumin (Biochrom AG, Berlin, Germany), 0.2% gelatin of cold-water fish skin (Sigma-Aldrich), 0.1% Triton X 100 (Carl Roth GmbH + Co. KG, Germany) and 2.5% normal horse serum (Vector Laboratories) before avidin (Vector Laboratories) was added. Afterwards, incubation was performed with primary antibodies overnight at 4˚C. The used antibodies with corresponding dilution are listed above (Table 2). The antibody diluent consisted of blocking buffer and biotin in PBS. On the next day, biotinylated secondary antibodies were applied and incubated for 30 min. Afterwards, the ABC-reagent (Vector Laboratories) was applied and incubated for 30 min. Antibody complexes were detected with 3,3′-diaminobenzidine (DAB) reagent (Vector Laboratories). Finally, counterstaining with hematoxylin was performed. The following positive controls served as quality assurance: human lymph-node metastasis of endometrial cancer tissue for IL-6 and SelK, human testis tissue for IL-1 alpha, GLB1, ALDH1 A1 and ALDH1 A3, embryonal rhabdomyosarcoma tissue for Pax7 and Myosin fiber type, human medulla oblongata tissue for CD56.

### Microscopy

An upright microscope (BX53; Evident, Hamburg, Germany), equipped with a motor-controlled table and a camera (DP28-CU; Olympus, Hamburg, Germany), was used. Images were processed using *cellSens* software (Olympus). The files were processed as.jpeg in ImageJ software and annotated.

In each section, a total of 75 muscle fibers, localized in three randomized areas (25 fibers per area), were captured at 20-fold magnification and analyzed for specific immunohistochemical antibody staining. A mean value of all analyzed areas represented one muscle tissue localization deriving from either intercostal muscle or diaphragm per patient. Quantified mean values of the same antibody staining from both localization per patient were combined and transferred into a total mean value representing an overall quantified mean value of antibody expression of one patient. To better illustrate the results, the stainings were grouped according to topic-related markers as follows: ALDH1 A1, ALDH1 A3, and SelK for oxidative stress; IL-1 and IL-6 for inflammation; CD56 and Pax7 as satellite cell markers; GLB-1 for cell senescence; and Myosin slow (ST) and Myosin fast (FT) for muscle fiber types. Mean values of quantified immunohistochemical antibody staining were classified into categories for the calculation of a representative mean value and declared as combined expression mean value.

### Statistical analysis

The study cohort was divided into CI-positive and CI-negative patients. Metadata of patients was analyzed in both groups and is presented by counts and percentages for categorical data and means or medians for continuous data. Linear regression analysis was applied to assess variables associated with topic-related markers. Further, a multivariable linear regression analysis including CI status, sex, and age was computed for each topic-related marker. All tests were two-sided and *p* values ≤ 0.05 were considered statistically significant. Statistical analyses were performed with R (version 4.3.1).

## Results

### Oxidative stress and inflammation

**Fig. 1:**
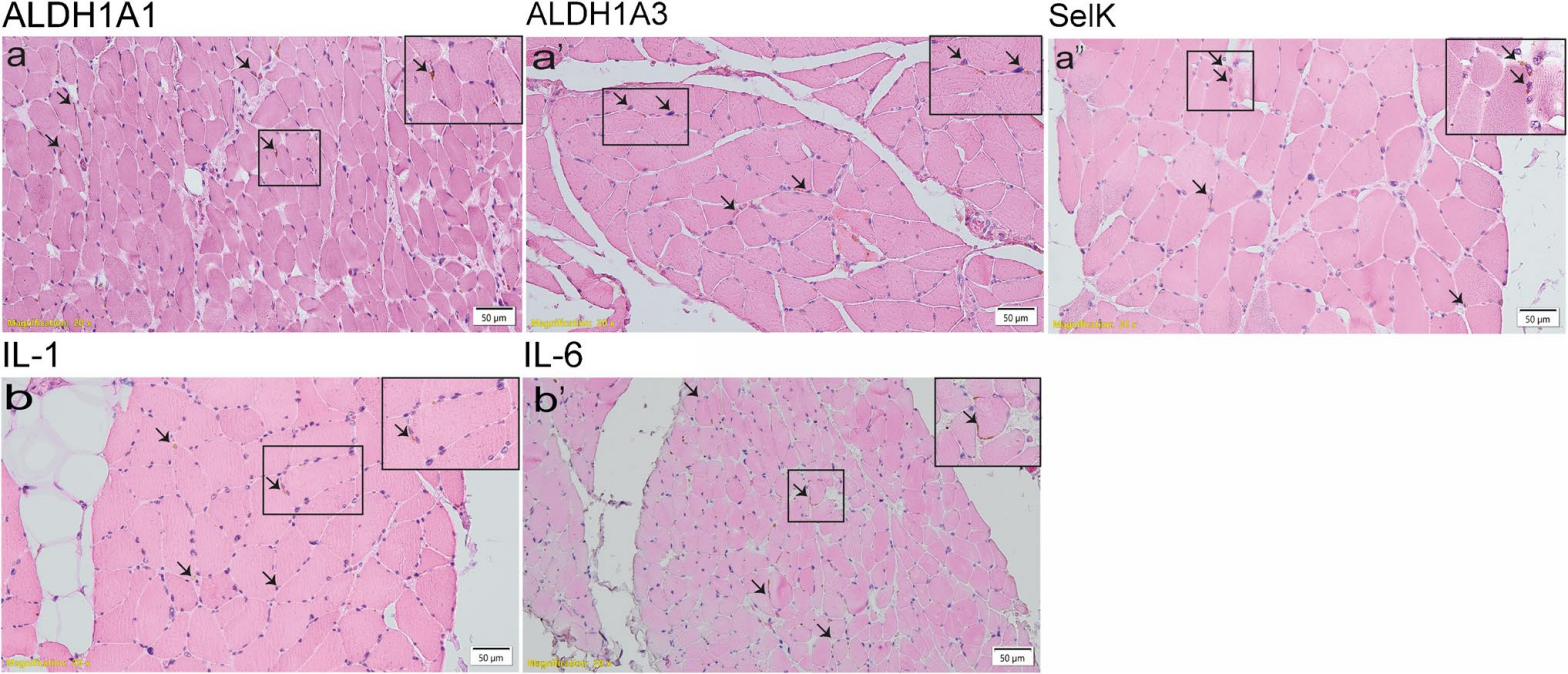
Representative images of oxidative stress (**a, a″**) and inflammation marker (**b, b′**) assessed by immunohistochemistry and eosin-counterstaining. Positive cells are indicated by arrows. Small insets display higher magnification of a selected region of the respective images. ALDH1 A1-positive cells in diaphragm of CI-positive patient (**a**). ALDH1 A3-positive cells in intercostal muscle of CI-negative patient (**a′**). SelK expression in diaphragm of CI-positive patient (**a″**). IL-1 (**b′**) and IL-6 (**b″**) in diaphragm of CI-positive patient. Scale bar: 50 µm

In intercostal muscle and diaphragm, marker of oxidative stress (ALDH1 A1, ALDH1 A3, and SelK), and inflammation (IL-1 and IL-6) displayed no difference in combined mean value of ALDH1 A1, ALDH1 A3 and SelK expression in CI-positive (8.13, SD = 0.3) compared to CI-negative patients (10.04, SD = 0.6; *p* = 0.12; Fig. 1a-a″, Suppl. Tab. 1a-d), and in combined mean value of IL-1 and IL-6 expression in CI-positive (28.5, SD = 3.3) compared to CI-negative patients (25.4, SD = 4.8; *p* = 0.44; Fig. 1b-b′, Suppl. Tab. 2 a-c).

### Satellite cells and cell senescence

**Fig. 2:**
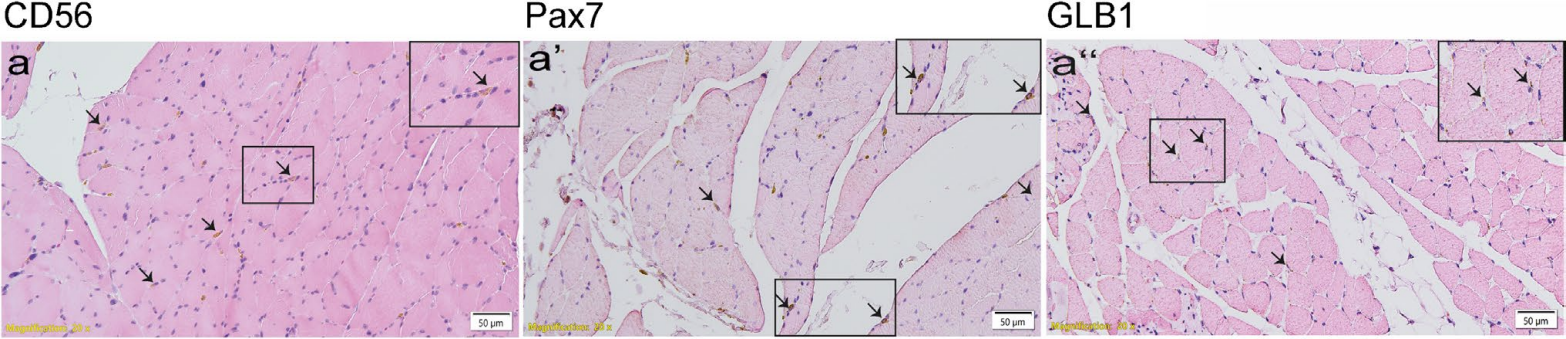
Representative images of satellite cell (**a, a′**) and cell senescence marker (**a″**), assessed by immunohistochemistry and eosin-counterstaining. Positive cells are indicated by arrows, respectively. Small insets display higher magnification of a selected region of the respective images. CD56-positive cells in intercostal muscle of CI-negative patient (**a**). Pax7-positive cells in intercostal muscle of CI-negative patient (**a′**). GLB1-positive cells in intercostal muscle of CI-positive patient (**a″**). Scale bar: 50 µm

In intercostal muscle and diaphragm, marker of satellite cells (CD56 and Pax7) displayed no difference in expression of CD56 (CI-positive: 2.56, SD = 1.712; CI-negative: 2.93, SD = 1.87; *p* = 0.57; Fig. 2a, Suppl. Tab. 3a) and Pax7 (CI-positive: 36.75, SD = 11.25; CI-negative: 30.13, SD = 14.29; *p* = 0.16; Fig. 2a′, Suppl. Tab. 3b) in CI-positive compared to CI-negative patients.

**Fig. 3:**
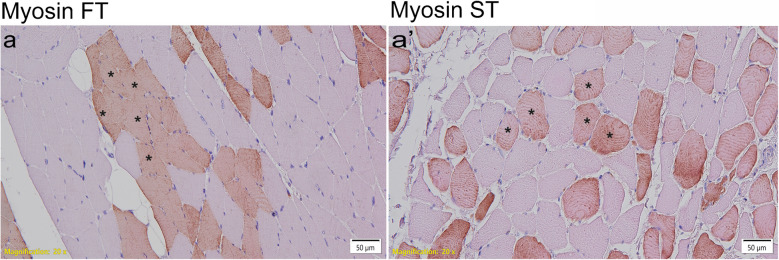
Representative images of muscle fiber type Myosin FT (**a**) and Myosin ST (**a′**), assessed by immunohistochemistry and eosin-counterstaining. Positive fibers are indicated by asterisk, respectively. Myosin FT-positive fibers in diaphragm of CI-negative patient (**a**). Myosin ST-positive fibers in diaphragm of CI-positive patient (**a′**). Scale bar: 50 µm

In intercostal muscle and diaphragm, senescent cell marker GLB-1 displayed no difference in CI-positive compared to CI-negative patients (CI-positive: 11.63, SD = 3.91; CI-negative: 11.80, SD = 6.46; *p* = 0.927; Fig. 3a″, Suppl. Tab. 3c).

### Muscle fiber type

In intercostal muscle and diaphragm, marker of muscle fiber type (Myosin FT: fast myosin, Myosin ST: slow myosin) displayed no difference in expression of Myosin FT (CI-positive: 12.66, SD = 2.18; CI-negative: 13.63, SD = 2.79; *p* = 0.28; Fig. 3a, Suppl. Tab. 4b) and Myosin ST (CI-positive: 12.34, SD = 2.18; CI-negative: 11.37, SD = 2.79; *p* = 0.28; Fig. 3a′, Suppl. Tab. 4a) in CI-positive compared to CI-negative patients.

### Correlation of ventilation status, patient-derived parameters, and clinical parameters

Basically, the study group was divided into ventilated compared to non-ventilated patients. The two groups were further graded to patient-derived (age, sex) and clinical parameters (CI status, type of in-clinic ventilation, times of COVID-19 vaccination, duration of hospitalization and lung-associated comorbidities (Table 3). No significant differences were found in patient derived metadata graded to ventilation-status (for *p* values, see Table 3). Moreover, the supply of ventilation was not conditional to the investigated clinical parameters (for *p* values, see Table 3).

**Table 3:** Statistical analysis of patient-derived (age, sex) and clinical (ventilation type, times of COVID-19 vaccination, duration of hospitalization, and lung-associated comorbidities) metadata, graded according to ventilation-application

Characteristics	Ventilated^1^, *N* = 16	Non-ventilated^1^, *N* = 15	*p*-value^2^
Age	MW: 71 (± 10), median: 68	MW: 74 (± 10), median: 77	0.383 …
Sex			0.213 …
Male	11 (61%) …	7 (39%) …	
Female	5 (38%) …	8 (62%) …	
CI status		…	0.210 …
Negative	6 (40%) …	9 (60%) …	
Positive	10 (63%) …	6 (38%) …	
Ventilation type			
Invasive	11 (100%) …	0 (0%) …	
Non	0 (0%) …	15 (100%) …	
NIV	5 (100%) …	0 (0%) …	
Times of COVID-19 vaccination			
2x	2 (100%) …	0 (0%) …	
3x	4 (40%) …	6 (60%) …	
4x	0 (0%) …	1 (100%) …	
Unvaccinated	7 (88%) …	1 (13%) …	
Unknown	3	7	
Duration of hospitalization	MW: 11 (± 11), median: 9	MW: 6 (± 8), median: 4	0.184 …. …
Unknown	0	3	
Lung-associated comorbidity			0.113 …
Yes	7 (78%) …	2 (22%) …	
No	9 (41%) …	13 (59%) …	

**Fig. 4:**
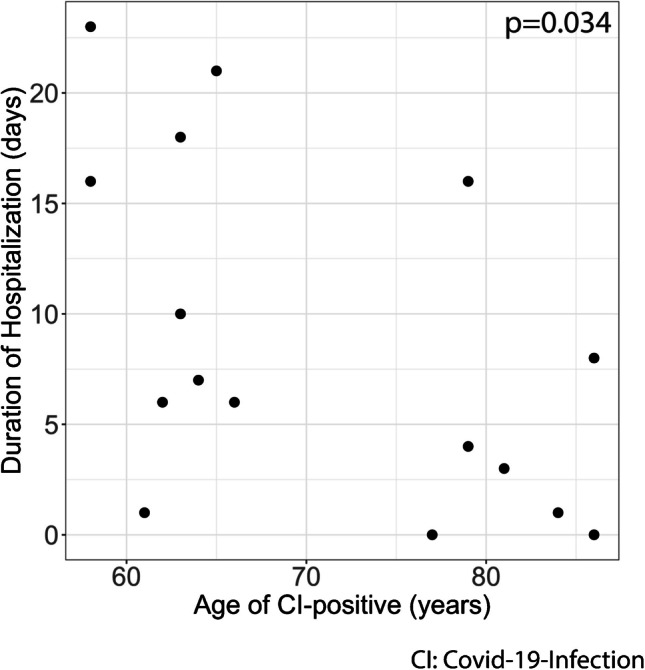
Correlation analysis between age of CI-positive patients and duration of hospitalization reveal a negative correlation between the two parameters (*p* = 0.034)

Interestingly, the age of CI-positive patients was negatively correlated with a longer duration of hospitalization (*p* = 0.034, Fig. 4).

### Correlation of RMD-related histopathology, patient-derived parameters, and clinical parameters

When adjusted to age and sex, CI-positive patients displayed higher number of PAX7-positive satellite cells when compared to CI-negative patients (PAX7 increased by 9.8 (95% CI: 0.5–19.2), *p* = 0.048, Table 4). No other correlations were observed between CI status and RMD-related histopathology.

**Table 4:** Correlation analysis between Pax7-positive satellite cells and CI-positive patients adjusted to age and sex (*p* = 0.048)

PAX7combined mean value	Estimate	Standard error	95% CI	*p*-value
CI (Ref.: negative)				
Positive	9.87	4.77	0.52 to 19.21	0.048
Age	0.35	0.22	− 0.09 to 0.79	0.128
Sex (Ref.: male)				
Female	5.83	4.82	− 3.62 to 15.27	0.237

**Fig. 5:**
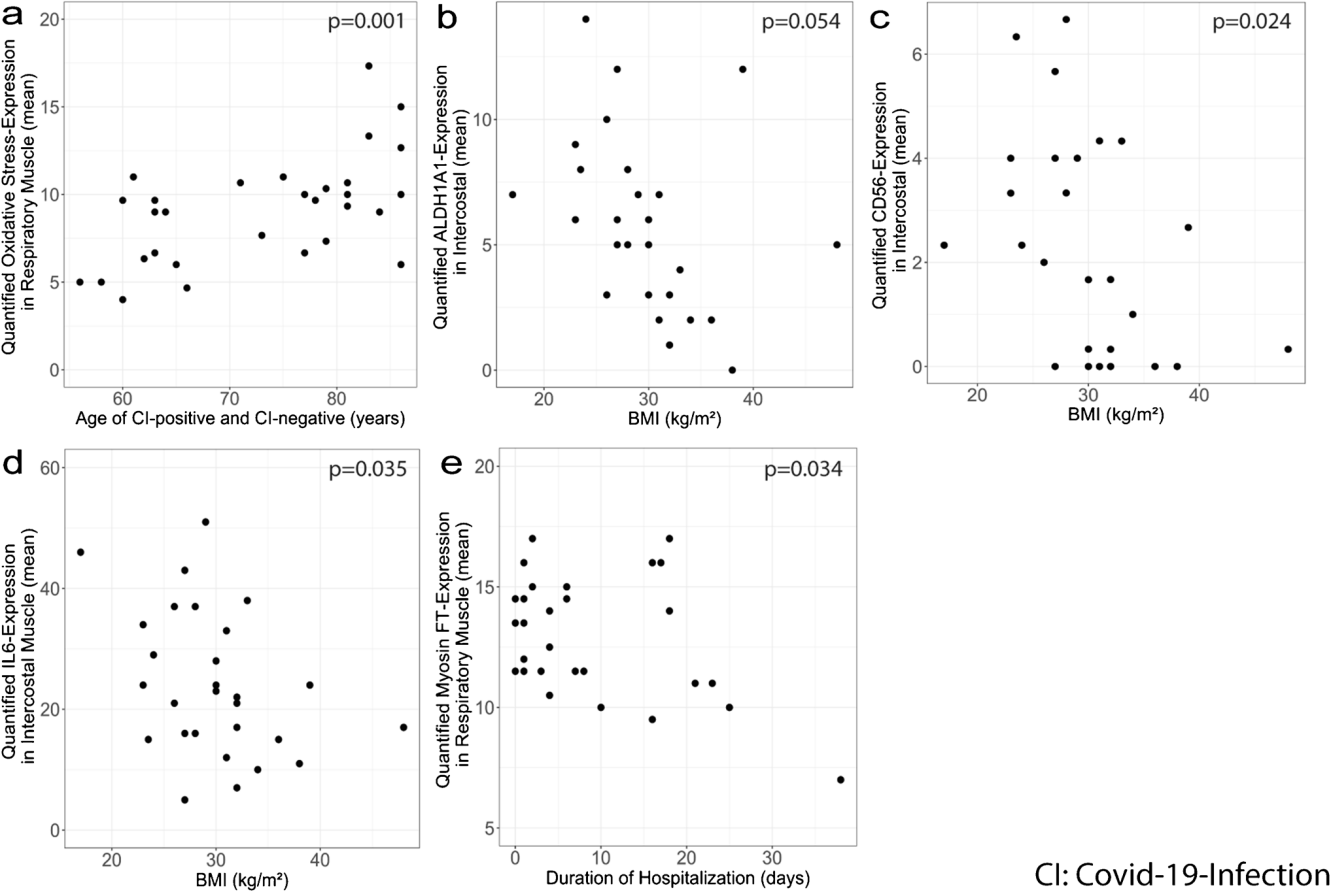
Correlation between RMD-related histopathology, patient-derived and clinical data. (**a**) Quantified oxidative-stress-expression in respiratory muscle and age (*p* = 0.001). **b** ALDH1 A1-expression in intercostal muscle and BMI status (*p* = 0.05). **c** CD56-expression in intercostal muscle and BMI status (*p* = 0.024). **d** IL6-expression in intercostal muscle and BMI status (*p* = 0.035). **e** Myosin FT-expression in respiratory muscle and duration of hospitalization (*p* = 0.034)

Independently from CI status, a correlation between RMD-related histopathology, patient-derived, and clinical parameters was observed. Related to age, older patients exhibit higher numbers of senescent cells in intercostal muscle and diaphragm (*p* = 0.027, estimate: 0.20 (95% CI: 0.03–0.37); data not shown). Results further outlined a positive correlation between age and oxidative stress markers in intercostal muscle and diaphragm tissue samples (*p* = 0.001; Fig. 5a).

In intercostal muscles of patients with high BMI, lower numbers of ALDH1 A1 positive cells were observed (*p* = 0.054; Fig. 5b). Patients with high BMI demonstrated lower numbers of CD56-positive satellite cells in intercostal muscle (*p* = 0.024; Fig. 5c). Inflammatory marker IL-6 was significantly prevalent in intercostal respiratory muscles of patients with low BMI compared to patients with high BMI (*p* = 0.035; Fig. 5d).

The study posed a negative correlation between hospitalization time the number of Myosin FT fibers (*p* = 0.034, Fig. 5e).

## Discussion

The present study was designed to investigate the influence of acute CI on RMD using postmortem histopathological examinations. We focused on markers of oxidative stress, inflammation, satellite cell status, cell senescence and muscle fiber composition, since these markers are commonly used to analyze satellite cell-dependent physiological and pathophysiological adaptations in skeletal muscles tissue (Dumont et al. 2015; Yin et al. 2013).

Contrary to our main hypothesis, we could not detect any direct correlation between CI status and the expression pattern of the histopathological markers used. This was surprising to us, because currently dyspnea in acute, long, and post-CI is attributed not only to pathophysiological processes in the respiratory tract but also in respiratory muscles (Severin et al. 2022; Silva et al. 2021). In this context, it must be noted self-critically that a methodological weakness of our study is the small number of patients and not always complete patient information such as the number of corona vaccinations. Only when adapted to age and sex, we observed increased numbers of cells labeled with anti-Pax7, a marker specific for activated, regenerative satellite cells (Rahman et al. 2023; Sincennes et al. 2021), in CI-positive patients, when compared to matched CI-negative controls. This could indicate that regenerative processes, including increased myogenesis, occur in respiratory muscles of acute CI-positive patients to counteract virus-induced intramuscular pathophysiological processes. However, we were unable to detect any other CI-associated differences in the histopathological examinations, using markers for oxidative stress, inflammation and muscle fiber type composition. In this regard, we cannot completely rule out that other methods in addition to histopathological analyses at light microscopic level, such as ultra-structural analyses using electron microscopy or molecular screening methods led to the detection of pathophysiological processes, or not. Of note, immunohistochemical analyses of parameters being associated with inflammation or oxidative stress only provide indications of putative pathophysiological processes, but do not comprehensively dissect mal- or dysfunctional mechanisms. A potential approach for an extended evaluation of the inflammatory response would be the retrospective analysis of documented blood test results. However, this approach only allows conclusions about overall body conditions and provides solely indirect insight into the effect of CI-related inflammation or oxidative stress in RMD. Further, a molecular analysis of respiratory muscle samples would be an alternative method to further examine the directed inflammatory responses but provided that unfixed material has been additionally collected. Moreover, fortunately, it is currently no longer possible to enlarge the cohort size of the present study to increase statistical power. Nevertheless, regarding long or post-CI, myopathy was identified as a cause of fatigue in long-term post-CI symptoms (Hejbøl et al. 2022). Referring, ultrastructural pathological changes in capillaries, nerves, and endomysium were observed by use of electron microscopy in 16 patients suffering from post CI, complaining about fatigue, myalgia or general weakness, indicating the re- and degenerating processes in respiratory muscles may occur in long- and post-CI.

Functional impairment of the lungs is often accompanied by disturbed neuromuscular transmission, both of which contributing to the severeness of this respiratory disease (Ali & Kunugi 2021; Farr et al. 2021). Respiratory skeletal muscle plays a crucial role in efficient lung ventilation and metabolic health treatment (Barber et al. 2025). In CI-positive patients, an accelerating sarcopenia with myopathic features of metabolic alterations and immune cell infiltration was observed when compared to CI-negative controls (Shi et al. 2021; Soares et al. 2022). There is, however, still controversy as to whether RMD is directly triggered by SARS-CoV-2, since viral infection itself may cause damage to the respiratory muscles (Severin et al. 2022). Potential direct viral toxicity could principally also occur in extra-pulmonal tissue expressing the receptor of the viral spike (S) protein, namely, angiotensin-converting enzyme 2 (ACE2; (Jackson et al. 2022)). By this, microvascular injury, immune system dysregulation and impairment of neuromuscular transmission might be underlying pathophysiological processes in RMD (Gupta et al. 2020; Nalbandian et al. 2021). However, in a hamster model of CI, infection induced myofiber atrophy and persistent energy metabolism suppression was observed, without direct viral invasion of the skeletal muscle tissue (Homma et al. 2024). Thus, it is likely that pathological effects may be induced by the combined systemic interferon and TNF-α responses at the acute phase and may contribute to post-CI persistent muscle fatigue.

Beside CI status, we also evaluated the relevance of patient-related and clinical metadata, such as age, sex, BMI, vaccination status, ventilation support, prevalent lung-associated comorbidities, and duration of hospitalization, in the further course of our investigations. In this regard, we were able to obtain fundamentally interesting and clinically relevant findings.

It is well known that ventilation support in intensive care units (ICU) is often associated with muscular weakness, which has a counterproductive effect on recovery in medium to long term (Vanhorebeek et al. 2020). Regarding care and treatment of acute CI, numerous studies showed that oxygenation supply or even invasive ventilation was necessary in the treatment of many severe cases (Butler et al. 2023). This naturally raised the question of whether the pathophysiological processes in the lungs that lead to respiratory distress are accompanied in the medium to long term by pathophysiological changes in the respiratory muscles, potentially induced by mechanical ventilation. As our findings demonstrate ventilation support is not conditional to the CI status or to lung-associated comorbidities, hence, future studies could emphasize the role of RMD as cause of dysfunctional respiration with focus on neuromuscular junctions.

The often-fatal outcomes and high number of patients requiring intensive care, including mechanical ventilation, ultimately led relatively quickly to the successful development of vaccines. Of note, the documentation of CI-vaccinations was not systematically collected for CI-negative patients in our study and therefore, not recorded in patient files of all investigated controls. As a result, no statistical analysis could be conducted to assess the potential positive impact of CI-vaccination on the necessity of ventilation support in ICU of investigated CI-positive compared to CI-negative patients.

Comorbidities such as obesity increase morbidity and mortality in CI-patients (Gao et al. 2021; Zhang et al. 2023). In distinction to a combined evaluation of clinical data with the histopathological analysis of respiratory muscle, we could reveal overall correlations with RMD: Patients with high BMI show a significant decrease of satellite cells and oxidative stress scavenger, with an accumulation of cytokines in intercostal skeletal muscle. This underlines the previously addressed characteristics of histopathological remodeling in skeletal muscle. Again, intercostal muscle demonstrates a correlation between BMI and histopathological remodeling processes. Furthermore, age adopts a crucial role in RMD-associated processes, since older subjects demonstrate a significantly major oxidative stress value and higher inflammatory response in both diaphragm and intercostal muscle. Interestingly, patients with extended hospitalization periods demonstrate a predominant Myosin FT ratio with no significant correlation with CI, age, or sex. Depending on the severity of the illness, patients undergo long durations of immobility, which promotes muscle fiber type degeneration (Ji & Yeo 2019) but also muscle tissue remodeling as observed in coma-patients (Lad et al. 2020). Of note, in case of positive CI-patients, previous studies describe a transition of slow to fast myosin fiber types by reason of CI-related mitochondrial dysfunction and reduced exercise capacity (Boer et al. 2022; Pleguezuelos et al. 2021), but so far, we could not validate this aspect. Nonetheless, these data highlight the need of combined analysis of clinical metadata and histopathology in context of RMD to draw a reasonable argumentation of histopathological processes.

In conclusion, our study addressed RMD in deceased CI-positive patients compared to controls with concomitant integration of patient-related and clinical metadata. Our histopathological investigations alone did not identify CI-dependent pathophysiological processes in diaphragm and intercostal muscle, but solely demonstrated increased numbers of active satellite cells when compared to controls. Interestingly, expression of markers representing satellite cell-associated physiological and pathophysiological processes in skeletal muscle tissue did not solely dependent on CI status but were associated with age, BMI, and prolonged duration of hospitalization. Based on this, our data generally indicate that a predisposed subgroup of patients needs to be identified in the respective clinical context, since distinct groups of patients are prevalent to onset and progression of RMD and as a consequence has to be assed with particular care. Interestingly, not only diaphragm but also intercostal muscle is affected by RMD in association to moderate and high BMI. Thus, intercostal muscle seems to be mostly impacted by RMD in patients with normal to adipose body composition and may be addressed with early breathing therapy to preserve respiratory function. Furthermore, we demonstrate that long durations of immobility are correlated with muscle fiber type transformation from Myosin ST to FT, which reflects a critical factor for tissue remodeling in respiratory skeletal muscle. According to immobility-dependent tissue remodeling, assessment of physical therapy may adequately activate the skeletal muscle fibers and diminish conditional transformation. Overall, our findings indicate that integration and evaluation of patient data, irrespective of the CI status, is recommended for meaningful analyses and interpretation of RMD associated histopathological analysis.

## Supplementary Information

Below is the link to the electronic supplementary material.

Supplementary file1 (JPG 386 KB)

Supplementary file2 (JPG 319 KB)

Supplementary file3 (JPG 311 KB)

Supplementary file4 (JPG 208 KB)

## Data Availability

No datasets were generated or analysed during the current study.
