# Supplementary figures and images for: Histopathological analysis of respiratory muscles in patients with acute COVID-19 infection
Source: Cell Tissue Res. 2025 May 5;401(1):59–68. doi: 10.1007/s00441-025-03973-3 (PMC12222437; doi:10.1007/s00441-025-03973-3)

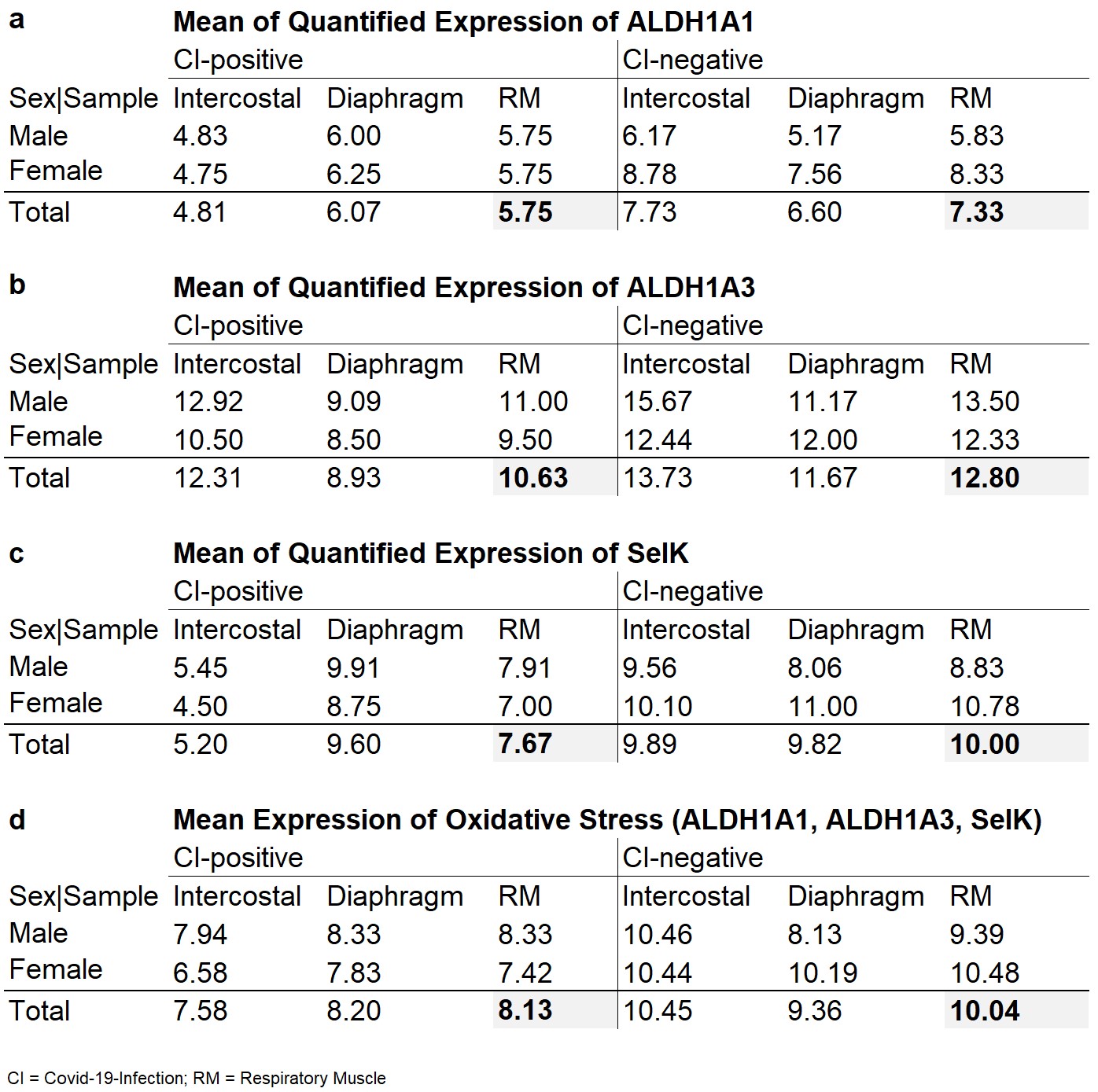

Supplement: Supplementary file 1 — Supplementary file1 (JPG 386 KB) [file 441_2025_3973_MOESM1_ESM.jpg]

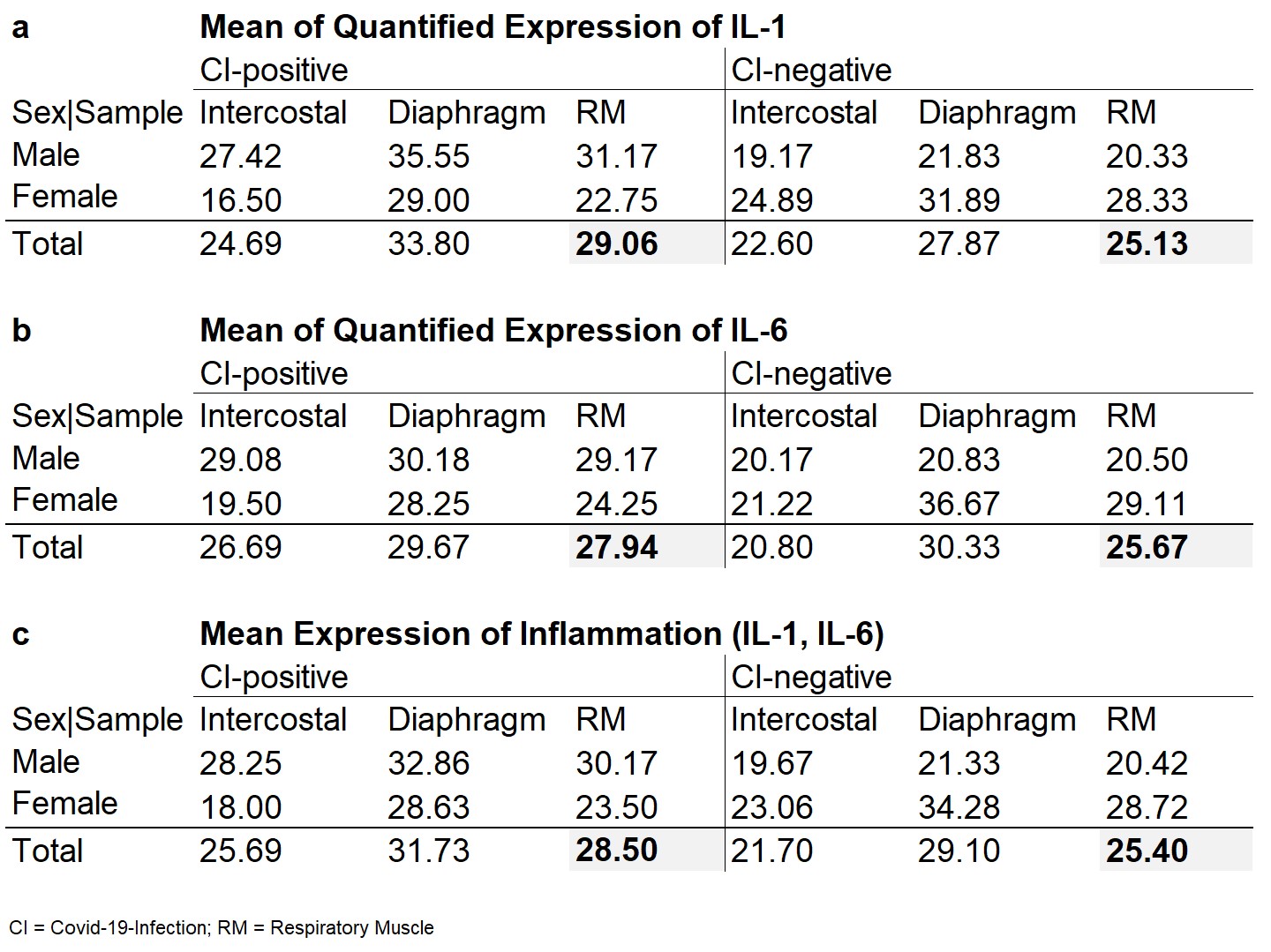

Supplement: Supplementary file 2 — Supplementary file2 (JPG 319 KB) [file 441_2025_3973_MOESM2_ESM.jpg]

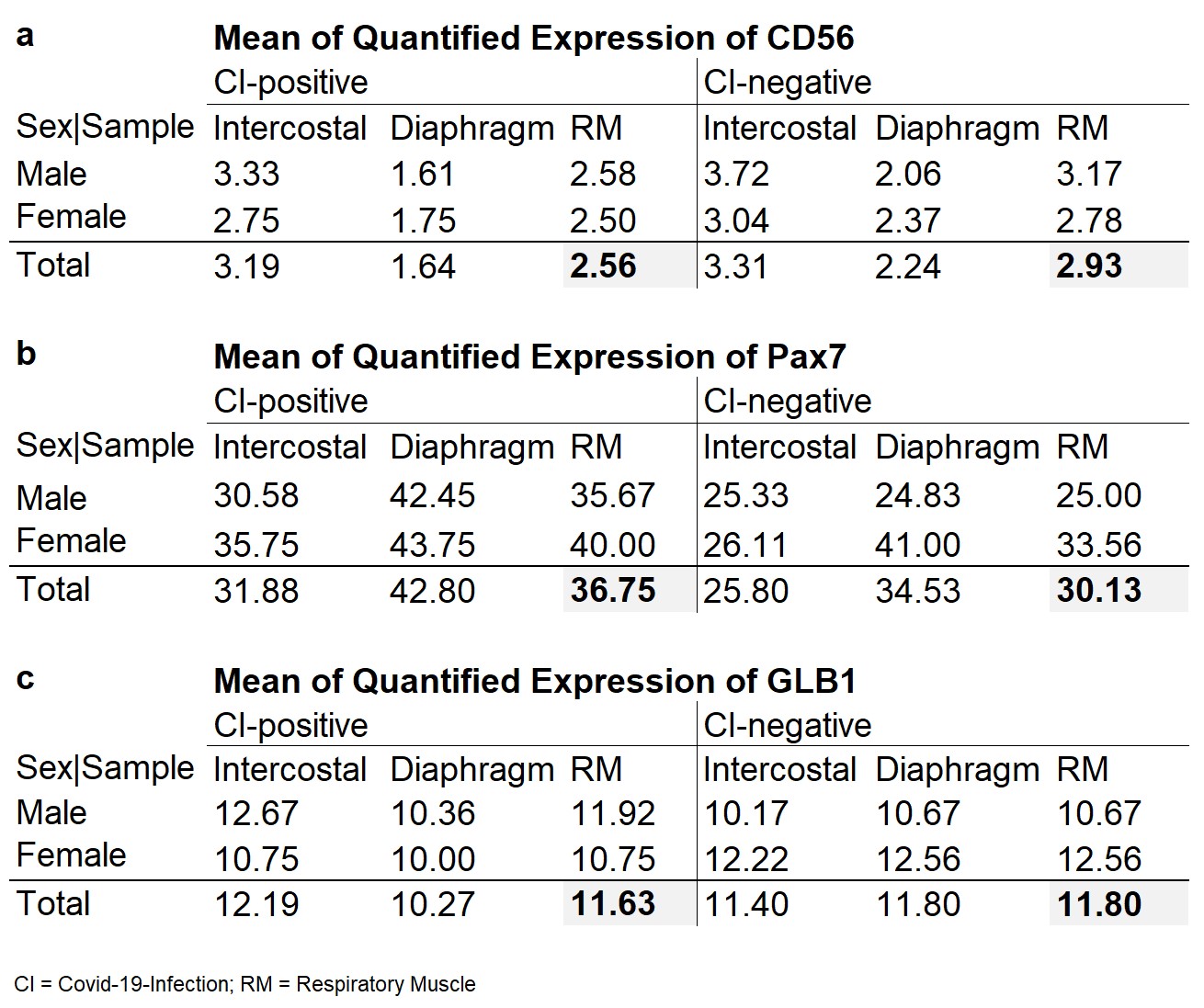

Supplement: Supplementary file 3 — Supplementary file3 (JPG 311 KB) [file 441_2025_3973_MOESM3_ESM.jpg]

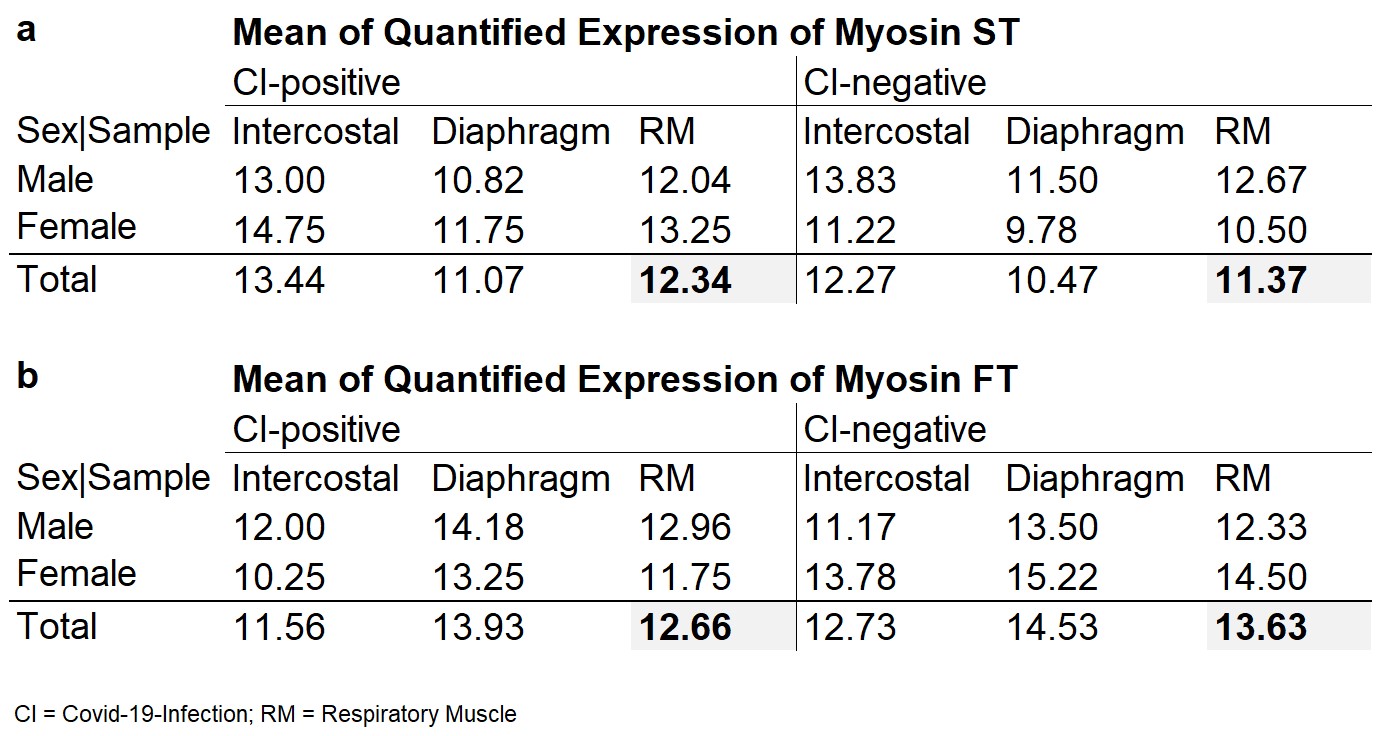

Supplement: Supplementary file 4 — Supplementary file4 (JPG 208 KB) [file 441_2025_3973_MOESM4_ESM.jpg]
